# Spatio-temporal analysis of malaria within a transmission season in Bandiagara, Mali

**DOI:** 10.1186/1475-2875-12-82

**Published:** 2013-03-01

**Authors:** Drissa Coulibaly, Stanislas Rebaudet, Mark Travassos, Youssouf Tolo, Matthew Laurens, Abdoulaye K Kone, Karim Traore, Ando Guindo, Issa Diarra, Amadou Niangaly, Modibo Daou, Ahmadou Dembele, Mody Sissoko, Bourema Kouriba, Nadine Dessay, Jean Gaudart, Renaud Piarroux, Mahamadou A Thera, Christopher V Plowe, Ogobara K Doumbo

**Affiliations:** 1 Department of Epidemiology of Parasitic Diseases, Faculty of Medicine and Dentistry, University of Sciences, Techniques and Technologies of Bamako, Point G, BP 1805, Bamako, Mali; 2 Howard Hughes Medical Institute/Center for Vaccine Development, University of Maryland School of Medicine, Baltimore, MD, USA; 3 Aix-Marseille University, Marseille, France; 4 Institut de Recherche pour le Développement, Montpellier, France

**Keywords:** Malaria, Geographic information system, Malaria transmission heterogeneity

## Abstract

**Background:**

Heterogeneous patterns of malaria transmission are thought to be driven by factors including host genetics, distance to mosquito breeding sites, housing construction, and socio-behavioural characteristics. Evaluation of local transmission epidemiology to characterize malaria risk is essential for planning malaria control and elimination programmes. The use of geographical information systems (GIS) techniques has been a major asset to this approach. To assess time and space distribution of malaria disease in Bandiagara, Mali, within a transmission season, data were used from an ongoing malaria incidence study that enrolled 300 participants aged under six years old”.

**Methods:**

Children’s households were georeferenced using a handheld global position system. Clinical malaria was defined as a positive blood slide for *Plasmodium falciparum* asexual stages associated with at least one of the following signs: headache, body aches, fever, chills and weakness. Daily rainfall was measured at the local weather station.

Landscape features of Bandiagara were obtained from satellite images and field survey. QGIS™ software was used to map malaria cases, affected and non-affected children, and the number of malaria episodes per child in each block of Bandiagara. Clusters of high or low risk were identified under SaTScan® software according to a Bernoulli model.

**Results:**

From June 2009 to May 2010, 296 clinical malaria cases were recorded. Though clearly temporally related to the rains, *Plasmodium falciparum* occurrence persisted late in the dry season. Two “hot spots” of malaria transmission also found, notably along the Yamé River, characterized by higher than expected numbers of malaria cases, and high numbers of clinical episodes per child. Conversely, the north-eastern sector of the town had fewer cases despite its proximity to a large body of standing water which was mosquito habitat.

**Conclusion:**

These results confirm the existence of a marked spatial heterogeneity of malaria transmission in Bandiagara, providing support for implementation of targeted interventions.

## Background

Malaria is one of the leading causes of morbidity and mortality in the world, with an estimated 3.3 billion people at risk of malaria [1]. The incidence of malaria worldwide is estimated to be 216 million cases per year, with 81% of these cases occurring in sub-Saharan Africa. Malaria kills approximately 655,000 people per year; 91% of deaths occur in sub-Saharan Africa [1], mostly in children under five years of age. In Mali, West Africa, malaria represents 36.5% of consultation motives in health center, it is a leading cause of morbidity and mortality children of less than five years of age and the first reason of anaemia in pregnant women [2]. Malaria transmission is seasonal.

Malaria parasite transmission and clinical disease are characterized by important microgeographic variation, often between adjacent villages, households or families [3-8]. This local heterogeneity is driven by a variety of factors including human genetics [9,10], distance to potential breeding sites [11,12], housing construction [2,13-16], presence of domestic animals near the household [17,18], and socio-behavioural characteristics [6,12,19,20]. WHO recommends the geographic stratification of malaria risk. An analysis of the local epidemiological situation is therefore essential, and such analyses formed one of the priorities of the 18th WHO Report [21], reiterated in the 20th WHO Report [22]. This involves an analysis of local variations, making it possible to define high-risk zones on a fine geographical scale, with the aim of increasing the efficacy of anti-malaria measures [23]. Setting up anti-malaria programmes targeting specific zones is therefore a priority. The development of Geographical Information Systems (GIS) has been an indispensable asset to this approach [24].

While seldom prioritized in the planning of malaria control by national programmes, the understanding of the microepidemiology of malaria is important to the design of effective small-area interventions [3,18], particularly in areas of unstable or very low transmission. To assess space-time local heterogeneity of disease, statistics that detect the presence of significant small-area disease clusters are often useful [2,7,25]. The space-time clustering of malaria has also been described, mainly in moderate to high transmission settings [2,13,26-30]. A few studies showed a difference of malaria risk at the regional or local level [27,31]. A precise knowledge of the geographic zones at risk, the levels of risk, the various risk factors, and the exposed populations, is required particularly in sites where malaria vaccines are tested. In order to assess space and time distribution of malaria disease in children in Bandiagara, Mali, within a transmission season, the data from a malaria incidence study have been used.

## Methods

### Study area

This space-time description of malaria distribution among children in Bandiagara, Mali is part of a multi-year cohort survey conducted by the Malaria Research and Training Center (MRTC) as part of the Bandiagara Malaria Project (BMP). This survey measures the age-specific incidence rates of clinical malaria episodes at a site dedicated to malaria clinical trials. Auxiliary parts of the project include molecular epidemiology studies, as well as *Plasmodium falciparum* genomic and transcriptomic analyses and serological investigations.

**Figure 1:**
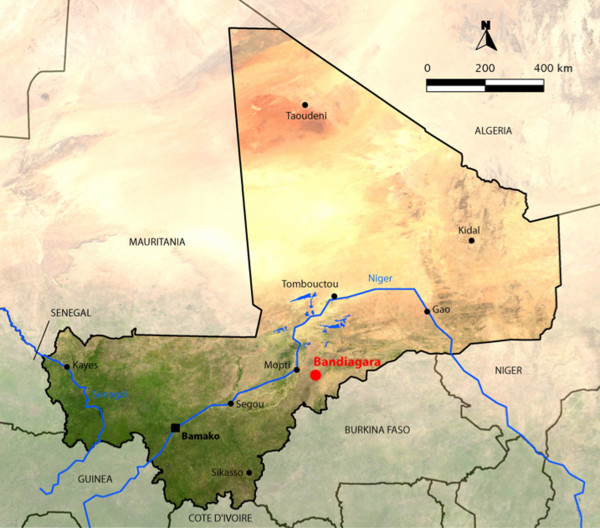
Map of Mali with the location of Bandiagara.

Bandiagara is a town, of approximately 13,364 inhabitants, situated in north-eastern Mali in West Africa (Figure 1) on a rocky plain above the Dogon escarpment and receiving a mean annual rainfall of 600 mm. The rainy season spans from June to October and the dry season from November to May. Bandiagara has a small river, the Yamé, a minor tributary of the Niger which stops flowing during the dry season, and transient post rainfall standing water body during the rainy season. *Anopheles gambiae* is the principal malaria vector and malaria transmission is highly seasonal meso- to hyperendemic [32].

With less than one infecting bite per person per month at the height of the dry season in March, the transmission season starts in June, peaks at up to 60 infected mosquito bites per person per month in August or September, and ends in December Following transmission fluctuations, malaria incidence is seasonal too, with an intense peak in September to October. In 1999, the clinical malaria incidence was 1.7 episodes per transmission season in children less than 10 years [33,34]. *Plasmodium falciparum* represents 97% of malaria infections, *Plasmodium malariae* 3%, and rare infections are due to *Plasmodium ovale*.

### Cohort description

This study was approved by institutional review boards of the Faculty of Medicine, Pharmacy and Dentistry of the University of Mali and of the University of Maryland School of Medicine. After obtaining permission to work in the community from local officials, elders, and traditional healers as described by Diallo *et al.*[35], the study was publicized by local radio broadcast, and parents were invited to accompany children aged up to six years to the BMP research clinic to be screened for eligibility. Children in the target age group were eligible for inclusion in the study if they met each of the following inclusion criteria: below six years of age at the time of screening, resident in Bandiagara town, good general health based on clinical evaluation, written informed consent obtained from the parent/guardian, and participation feasible through the 48-month follow up. Exclusion criteria were: simultaneous participation in an interventional clinical trial, chronic medication with known anti-malarial activity (such as trimethoprim-sulphamethoxazole for prevention of AIDS-associated opportunistic infections), or any condition that in the opinion of the principal investigator would jeopardize the safety or rights of a participant in the trial or would render the participant unable to comply with the protocol.

Active and passive surveillance were conducted to capture the incidence of malaria infection and disease. Active surveillance consisted of scheduled monthly visits aimed at detecting asymptomatic malaria infection and anaemia. Clinical examination of the participants was performed by the study physician at enrolment and on a quarterly basis. Following standard protocols, finger-prick blood samples were collected monthly for malaria smears, measurement of haemoglobin level, and parasite genotyping from filter paper. Venous blood was collected quarterly for molecular and immunological analyses. Passive surveillance consisted of continuous availability of free, expeditious, high quality, basic medical care at the BMP research clinic and Bandiagara District Hospital, where parents/guardians were instructed to consult whenever their child was sick. Children were then examined by a physician, and axillary temperature was checked (fever was defined as axillary temperature ≥37.5°C). Blood samples were collected for microscopic examination (thick blood film), haemoglobin level determination, and parasite genotyping. Malaria was treated with artemisinin combination therapy (artesunate + amodiaquine or artemether + lumefantrine) according to the guidelines of the Mali malaria control programme.

### Malaria data, rainfall data, and Bandiagara GIS

This open cohort was enrolled in June 2009 and will be followed up until July 2014. For the purpose of this study, the analysis was focus on the new malaria infections recorded during the first year of follow up (June 2009 to May 2010). Clinical malaria was defined as the association of a new positive thick blood smear with asexual *P. falciparum* parasitaemia and symptoms generally consistent with malaria (headache, body aches, fever, chills, or weakness, irrespective of body temperature at the time of examination). After aggregation on a weekly time scale, time series of the number of malaria episodes was plotted together with rainfalls measured at the local weather station in Bandiagara. By segregating the first and second parts of the rainy and dry seasons, the global time series was then divided into successive periods.

At inclusion and in case of relocation, the household of each child (i e, the place where the child slept) was georeferenced using a handheld global position system (Garmin® Personal Navigator; accuracy approximately within 10 m).

Children household and malaria episodes occurrence were mapped and a Geographical Information System was developed for the study area that also included the Bandiagara house blocks and the water bodies of the area based on a satellite image (Quick Bird, August 2004) and field surveys (2010).

With the demographic expansion of the town and a flood in July 2007 that destroyed many houses in quarters 1, 2, and 5 on the right bank of the Yame River, new neighbourhoods have been built in the north and east. Because no updated satellite image was available at the time of the present study, house blocks of arbitrary shape and size were drawn for the few included children living in these new quarters.

### Case mapping and spatial statistical analysis

Using Quantum GIS™ software (QGIS™) version 1.7.3 [36], children households were plotted according to their geographic coordinates. Numerous children, likely siblings of the same family, shared the same location. For each corrected location, data were subsequently aggregated, and several variables were calculated: initial number of study participant, total and daily number of recorded malaria episodes, daily number of susceptible children (taking into account the excluded children and a three-week refractory period after a malaria episode). After aggregating data at the house block level, the mean number of malaria episodes per child was calculated for each block over the entire year. The mean number of malaria episodes per child-week was also calculated for each successive period.

Using QGIS™, the numbers of included children asnd malaria episodes were mapped using proportional circles. The spatial distribution of malaria risk was illustrated by choropleth mapping at the block level of the mean number of malaria episodes per child, as described above.

In order to better assess the spatial variability of malaria risk, a cluster analysis was performed using Kulldorff’s statistics through the SaTScan® software [25,37]. This widely applied method [5,38-40] moves a circular or elliptic scanning window over the study area and compares observed and expected case numbers inside and outside this window in order to detect clusters and estimate risk ratios. Using daily malaria episodes and susceptible children at each location, a Bernouilli distribution model with 50% of the population at risk, and elliptic scanning windows, high or low risk purely spatial clusters were sought over the whole year and over each study period. The standardized incidence ratio (SIR) was defined as the ratio of observed to expected cases. Cluster significance (*P*-value) was computed with a likelihood ratio test provided by a Monte Carlo approach using 999 random simulations under the null hypothesis of no cluster. Statistically significant spatial clusters (*P*-value < 0.05) were subsequently mapped on QGIS™.

## Results

### Spatial distribution of sampled children and recorded malaria episodes

**Figure 2:**
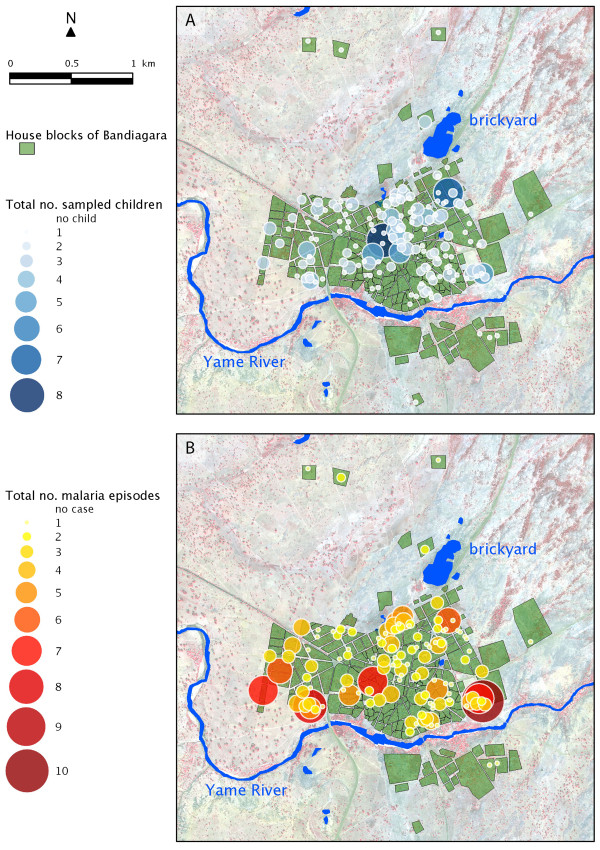
Corrected spatial distribution of (A) the 300 children sampled, and (B) the 296 malaria episodes recorded between June 2009 and May 2010 in Bandiagara.

Study children lived in 168 locations which have been were geopositioned. Children habitats were mainly distributed in the centre and north-eastern blocks of Bandiagara (Figure 2A).

During the course of the study period, 11 active surveys were carried out, and a total 296 *P. falciparum* clinical episodes were recorded among 178 children. Fifty-one episodes were documented from active surveys and 245 episodes from passive survey. Episodes mostly concentrated along the right banks of the Yamé River and, to a lesser extent, on the north-eastern blocks of the city near the brickyard (Figure 2B), exhibiting a marked spatial heterogeneity.

### Malaria episodes and rainfall times series

**Figure 3:**
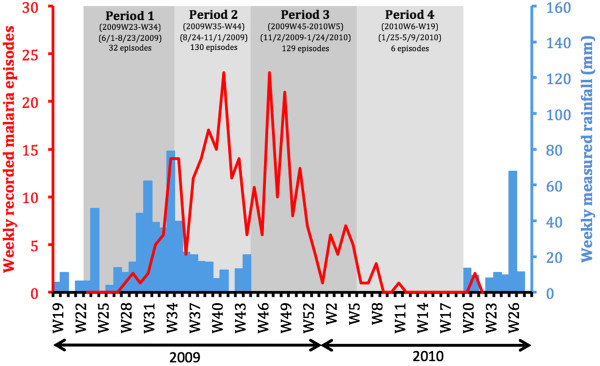
Recorded malaria episodes and measured rainfall weekly time series.

In order to analyse the temporal distribution of malaria episodes, data were aggregated by week, and a time series of malaria episodes was plotted together with locally measured rainfalls (Figure 3). In 2009, the first malaria clinical episodes had a lag of four weeks after the onset of the rainy season. Children continued to experience malaria episodes late into the 2009–2010 dry season (from December 2009 to May 2010), a period that accounted for nearly half of the 296 total episodes. With respect to the rainfall time distributions, four successive periods were delimited (Figure 3). Period 1 extended from the beginning of the study (2009 week 23) until the rainfall maximum (2009 week 34; 1 June to 23 August 2009). Period 2 spanned the rest of the rainy season (from 2009 week 35 to week 44; 24 August to 1 November 2009). Period 3 covered the first half of the dry season (2009 week 45 to 2010 week 5; 2 November 2009 to 24 January 2010). Period 4 extended throughout the second half of the dry season (2010 week 6 to week 19). Because this was the start of the next rainy season, the remaining period in the time series (2010 week 20 to week 23) was excluded from further period-based analyses.

### Malaria episodes per child

**Figure 4:**
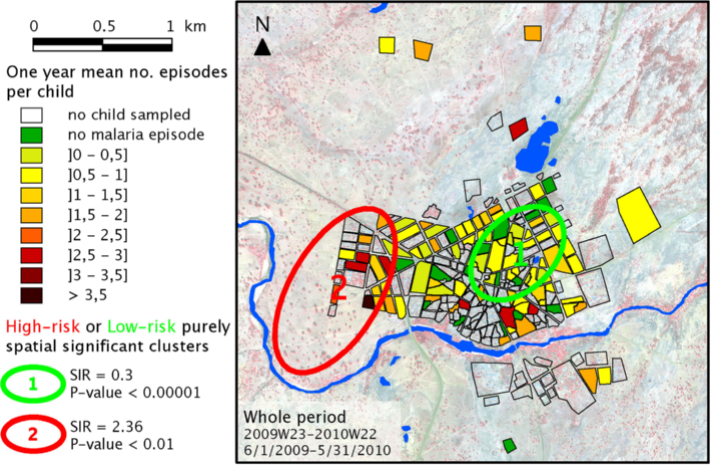
**Number of malaria episodes per sampled child over the full one-year period.** Localization of the significant high-risk or low-risk spatial clusters computed on SaTScan®.

In order to better visualize the malaria risk across Bandiagara and to minimize any sampling bias, the mean number of malaria episodes recorded per sampled child was mapped for each block (Figure 4). Over the entire year of follow up, while the greatest risk of malaria transmission seemed to be located on the western blocks, along the northern shore of the Yamé River and on the northern side of the brickyard, the centre of the town exhibited a patchy yet globally low risk pattern.

**Figure 5:**
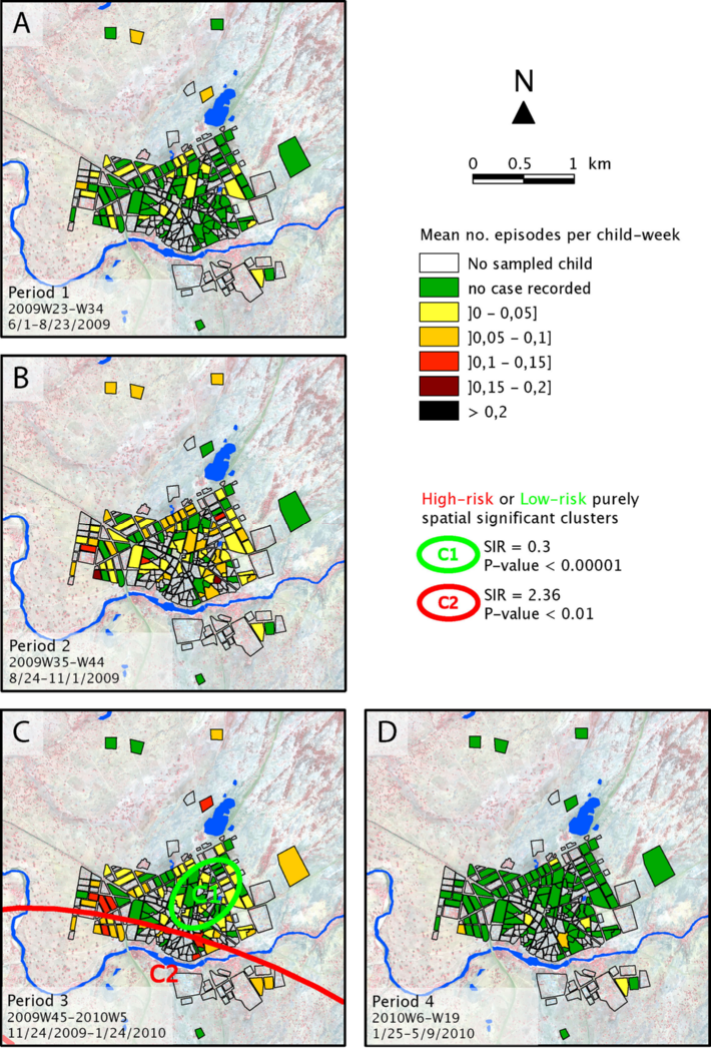
**Number of malaria episodes per sampled child-week over each of the four periods.** Localization of the significant spatial clusters computed on SaTScan®.

For each of the four epidemiological periods, as defined in Figure 3, a similar choropleth mapping of the mean number of episodes per child-week was computed, as illustrated in Figure 5. These sequential maps exhibited a contrasting pattern. While malaria incidence appeared globally low during the beginning of the rainy season (Period 1, Figure 5A), especially in the centre of Bandiagara, malaria incidence was much higher during the end of the rains (Period 2, Figure 5B), with a patchier distribution. During the first half of the dry season, incidence became more intense on the edge of the town, particularly along the Yamé River and just north of the brickyard (Period 3, Figure 5C). Malaria risk returned to negligible during the second part of the dry season (Period 4, Figure 5D), except for a few limited foci in the southern part of the town.

### High or low-risk spatial clusters

To statistically confirm this spatial distribution of malaria risk across Bandiagara, high or low-risk spatial clusters were sought using Kuldorff’s statistics. In the full year of study (Figure 4), the first significant cluster was a low-risk cluster located on the eastern part of the town centre and separated from the brickyard and the Yamé River by a few blocks of houses (SIR = 0.3; *P*-value <10^-5^). A secondary significant high-risk cluster covered the western blocks of Bandiagara located near the bend of the Yamé River (SIR = 2.36; *P*-value <0.01). During Period 1 (Figure 5A), this method didn’t establish any significant cluster, but a nearly significant low-risk spatial cluster included the south-east part of the town on both sides of the Yamé River (SIR = 0.091; *P*-value = 0.07). During Period 2 (Figure 5B), no area with a significantly higher or lower incidence could be detected. During the first half of the dry season (Period 3, Figure 5C), a significant low-risk cluster covered the eastern part of the town centre (C1, SIR = 0.3; *P*-value <10^-5^). A secondary high-risk cluster spanned most blocks located along the river (C2, SIR = 2.36, *P*-value <0.01). Conversely, no significant low-risk cluster was detected during Period 4 (Figure 5D).

## Discussion

The present work represents the first description of malaria local spatial microepidemiology. It constitutes a critical step in the temporal and spatial stratification of the local malaria risk, as recommended by WHO [22].

Following 300 children from June 2009 to May 2010, 296 clinical malaria episodes were recorded among 178 participants. Their occurrence exhibited a marked seasonal pattern that is typical in Sahelian regions where malaria transmission is unstable [41]. However, the peak of malaria incidence in Bandiagara, where malaria is meso- to hyperendemic, occurred several weeks after rainfalls and persisted late in the dry season. In relation to the dynamics of *Anopheles* breeding sites, such a pattern has been previously described in southern Mali (Doumbo, pers comm).

Breeding site location also likely explains the marked spatial heterogeneity of malaria incidence observed across Bandiagara in this year of follow up, similar to that previously described in Kenya by Midega *et al.*[42]. Most affected house blocks were located not far from the Yamé River, especially its bend west of the town, or near the brickyard. The increased malaria incidence of house blocks in the northern limit of the study area may have been related to a small backwater pond located in their vicinity. Conversely, the centre of Bandiagara appeared protected, likely thanks to a barrier effect of the households living in the blocks along the brickyard and the Yamé River such effect has previously been described by Gaudart [26].

The evolution of the spatial distribution of malaria risk in subsequent periods of the year may also denote the changing location of principal breeding sites. During the beginning of the rainy season, the relatively protected area in the south-east of Bandiagara could reflect the absence of breeding sites in the rapidly flowing Yamé River, while the brickyard fills with water. At the same time, numerous water holes appear on the rocky plateau north of the town. The end of the rainy season is thus characterized by widespread malaria transmission, explaining the absence of detectable clusters. On the contrary, although transmission remains high during the beginning of the dry season, the north-eastern blocks of the town appear protected, potentially because of increasing pollution of urban breeding sites, while transmission concentrates along the Yamé River, whose lesser flow with standing pools may allow mosquito breeding, and near the brickyard,

These hypotheses should be confirmed by further studies with longer time series as well as transmission and entomological surveys. This study proves the feasibility of thorough epidemiological studies in rural African areas. It also presents valuable tools to better understand malaria dynamics in endemic foci like Bandiagara, better target control interventions, and better design future clinical trials.

The lack of environmental factors, the limited study period and the time lag between satellite image, the lack of cut-off for temperature and parasitemia represent the limitations of this study. The implementation of additional investigations is therefore essential to take in account these items in an in-depth description of the micro epidemiology of malaria in Bandiagara.

## Conclusion

Despite its limitations which are the no taking in account the environmental factors, the follow up time limitation, the present work provides valuable information on the local distribution patterns of malaria in Bandiagara. These results confirm the existence of a marked spatial heterogeneity of malaria transmission, likely related to seasonal breeding sites.

## Abbreviations

AIDS: Acquired immune deficiency syndrome; BMP: Bandiagara malaria project; GIS: Geographical information system; MRTC: Malaria research and training center; SIR: Standardized incidence ratio; WHO: World Health Organization.

## Competing interests

The authors declare that they have no competing interests.

## Authors’ contributions

DC, MT, ML, BK, MAT, ND, JG, CVP and OKD were involved in the conceptualization, research design, data collection and preparation of the manuscript. YT, AKK, KT, AG, ID, AN, MD, AD and MS contributed significantly to study execution and data collection. SR, ND, JG and RP collaborated on the geographical data analysis, the mapping, and preparation of the manuscript. All authors read and approved the final manuscript.
